# MAGIC Study: Aims, Design and Methods using SystemCHANGE™ to Improve Immunosuppressive Medication Adherence in Adult Kidney Transplant Recipients

**DOI:** 10.1186/s12882-016-0285-8

**Published:** 2016-07-16

**Authors:** Cynthia L. Russell, Shirley Moore, Donna Hathaway, An-Lin Cheng, Guoqing Chen, Kathy Goggin

**Affiliations:** School of Nursing and Health Studies, University of Missouri-Kansas City, Health Sciences Building 2407, Kansas City, MO 64108 USA; Case Western Reserve University, 10900 Euclid Avenue, Cleveland, OH 44106 USA; Department of Advanced Practice and Doctoral Studies, 920 Madison, #924, Memphis, TN 38163 USA; Department of Internal Medicine, University of Kansas Medical Center, 4043 Wescoe, MS 1037 3901 Rainbow Blvd, Kansas City, KS 66160 USA; Health Services and Outcomes Research, Children’s Mercy Hospitals and Clinics, University of Missouri - Kansas City Schools of Medicine and Pharmacy, 2401 Gillham Road, Kansas City, MO 64108 USA

**Keywords:** Adherence, Randomized controlled trial, Outcomes, Transplantation, Cost-effectiveness

## Abstract

**Background:**

Among adult kidney transplant recipients, non-adherence to immunosuppressive medications is the leading predictor of poor outcomes, including rejection, kidney loss, and death. An alarming one-third of kidney transplant patients experience medication non-adherence even though the problem is preventable. Existing adherence interventions have proven marginally effective for those with acute and chronic illnesses and ineffective for adult kidney transplant recipients. Our purpose is to describe the design and methods of the MAGIC (Medication Adherence Given Individual SystemCHANGE™) trial

**Methods/Design:**

We report the design of a randomized controlled trial with an attention-control group to test an innovative 6-month SystemCHANGE™ intervention designed to enhance immunosuppressive medication adherence in adult non-adherent kidney transplant recipients from two transplant centers. Grounded in the Socio-Ecological Model, SystemCHANGE™ seeks to systematically improve medication adherence behaviors by identifying and shaping routines, involving supportive others in routines, and using medication taking feedback through small patient-led experiments to change and maintain behavior. After a 3-month screening phase of 190 eligible adult kidney transplant recipients, those who are <85 % adherent as measured by electronic monitoring, will be randomized into a 6-month SystemCHANGE™ intervention or attention-control phase, followed by a 6-month maintenance phase without intervention or attention. Differences in adherence between the two groups will be assessed at baseline, 6 months (intervention phase) and 12 months (maintenance phase). Adherence mediators (social support, systems-thinking) and moderators (ethnicity, perceived health) are examined. Patient outcomes (creatinine/blood urea nitrogen, infection, acute/chronic rejection, graft loss, death) and cost effectiveness are to be examined.

**Discussion:**

Based on the large effect size of 1.4 found in our pilot study, intervention shows great promise for increasing adherence. Grounded in the socio-ecological model, SystemCHANGE™ seeks to systematically improve medication adherence behaviors by identifying and shaping routines, involving supportive others in routines, and using medication taking feedback through small patient-lead experiments to change and maintain behavior. Medication adherence will be measured by electronic monitoring. Medication adherence persistence will be examined by evaluating differences between the two groups at the end of the 6-and 12- month phases. Mediators and moderators of medication adherence will be examined. Patient outcomes will be compared and a cost-effectiveness analysis will be conducted.

**Trial registration:**

ClinicalTrials.gov Registry: NCT02416479 Registered April 3, 2015

## Background

For adults who have a kidney transplant, the leading predictor of rejection, kidney loss, death and their attendant costs is immunosuppressive medications non-adherence [1–5] with an alarming one-third of kidney transplant recipients experiencing this preventable problem [6–8]. According to meta-analysis, predictors of medication nonadherence are nonwhite ethnicity, poorer social support and poorer perceived health [8]. Patients’ most frequent barrier to adhering to immunosuppressive medication is forgetting [9]. Even minor deviations from adherence have shown negative effects, though the precise extent of poor outcomes stemming from nonadherence is not yet clear [10–13]. Traditionally, intervention studies have aimed at boosting adherence target cognition (knowledge, attitudes, beliefs) and behavioral skills. However, these have proven only marginally effective for individuals with acute and chronic illnesses [14–18] and ineffective for adult kidney transplant recipients [19–21]. In a sample of kidney transplant recipients, we test the innovative and successful SystemCHANGE intervention, which is grounded in the Socio-Ecological Model [22–25]. This approach is a paradigm shift in behavioral interventions because it focuses on redesigning the system of the interpersonal environment and daily routines linked to health behavior, rather than focusing on increasing individuals’ motivation and intentions to improve their adherence [22, 26, 27]. Using a four-pronged, patient-centered approach, we: (1) assess individual systems (including important others who shape medication taking), how they influence medication taking and the individual’s proposals for improving medication adherence, (2) implement the proposed individual systems solutions for improving adherence, (3) track adherence data, and (4) evaluate adherence data through small experiments. The effect size of 1.4 found in the SystemCHANGE pilot work was a nearly four-fold greater effect size of most other previous adherence interventions [28].

The effectiveness of interventions to improve medication adherence (MA) in the acute and chronically ill general population has been examined by numerous systematic reviews and meta-analyses [14–16, 29–35]. Typically psychological theories guide interventions to enhance knowledge through education, attitude through counseling, and behavior through skills training. Even with multi-faceted interventions, effect sizes in meta-analyses have been very small. Narrative reviews corroborate findings from meta-analysis that limited benefits occur with interventions focused on motivation and intention. Only about 50 % of studies found statistically significant improvements in MA. Equally disappointing results have been noted in transplant intervention studies which have also focused only on motivation and intention [19–21, 36–40]. Limitations of these studies included: 1) atheoretical approaches, 2) testing interventions that targeted motivation and intentions, 3) a lack of attention to environmental influences on routines and habits, 4) a lack of timely feedback on medication-taking, and 5) no evaluation of intervention cost-effectiveness. This study protocol addresses these limitations.

## Methods

### Aims

The primary aim of the trial is to determine whether the SystemCHANGE™ intervention is more effective than the attention control intervention in improving MA in adult kidney transplant recipients at the completion of the intervention and maintenance phases. We hypothesize adult kidney transplant recipients participating in the SystemCHANGE™ intervention will have higher immunosuppressive MA rates than those participating in the attention control at the completion of intervention and maintenance phases. A secondary aim is to examine the patterns of MA in this group. We will determine when the intervention becomes effective (e.g., what “dose” is needed) and what the pattern of decay in medication adherence is over time in both groups. Our exploratory aims are to determine whether the SystemCHANGE™ intervention is more effective than the attention control in decreasing poor health outcomes (e.g., infection, acute/chronic rejection, graft loss, death, and increasing creatinine/blood urea nitrogen,), to explore potential mediators (social support and systems-thinking) and moderators (ethnicity, perceive health and level of medication nonadherence) of MA, and to determine whether the SystemCHANGE™ intervention is cost-effective. We hypothesize patients in the SystemCHANGE™ intervention will demonstrate lower levels of poor outcomes than attention controls at 12 months. We also hypothesize the cost-effectiveness ratio for the SystemCHANGE™ intervention will be less than the cost-effective ratio for the attention-control intervention.

## Design

This is a 4-year, two-center, randomized controlled trial, that is single-blind (participants [Pps]) and uses a stratified sample block design with repeated measures. We are comparing the SystemCHANGE™ intervention to the attention control intervention in adult kidney transplant recipients with existing medication nonadherence documented by electronic monitoring. The repeated-measures design provides longitudinal data regarding medication nonadherence which allows us to determine when the intervention becomes effective (to determine if a lower dose of SystemCHANGE™, e.g. shorter time of delivery, is possible). It also allows us to track possible decay in medication nonadherence over time following the intervention.

We are examining the experimental effect on the outcome variable MA. During the 3-month screening phase, all Pps are using electronic monitoring to document medication taking. Those who are adherent (MA rate of .85 % or greater) exit the study. To prevent the “ceiling” effect, those with documented medication nonadherence (MA rate of less than .85 %) are stratified by low (<70 %), and moderate (70-84 %) nonadherence, based upon our previous medication nonadherence pattern research [41]. They then enter the intervention phase of the study and are randomized into either the treatment (SystemCHANGE™ intervention) or the attention control group (attention control condition). During the 6-month intervention phase, all Pps receive a home visit at baseline plus six telephone calls (at intervention months 1, 2, 3, 4, 5, 6). In addition, Pps randomized into the SystemCHANGE™ intervention are also guided in implementing SystemCHANGE™ activities related to medication taking by the Research Assistant (RA). Control group Pps receive RA-provided education guided by healthy living patient educational materials. The maintenance phase begins after the intervention and runs for an additional 6 months. This phase examines how Pps maintain MA in the absence of an intervention; however, we are continuing to use electronic monitoring to measure the outcome variable. Health outcome and healthcare cost data are collected during the intervention and maintenance phases.

### Ethics, consent and permissions

Institutional Review Board (IRB) approval has been obtained at the University of Missouri and the University of Tennessee. The IRB approval at the University of Missouri, which is the primary approving institution, is #1210944. Informed consent is obtained from every participant prior to their involvement in the study. We are collecting demographic data from those who do not consent to the full study, but who agree to provide this information. This allows us to determine if any demographic differences exist between those who decline to participate in the study and those who consent.

### Conceptual model

‘To Err Is Human’, the Institute of Medicine’s landmark report on improving hospital safety, suggests moving away from blaming the individual and instead making the desired behavior more likely to occur by removing barriers [37]. SystemCHANGE™ is consistent with moving away from the culture of “blame” and instead guiding Pps to change their individual personal environment [38]. Additionally, sustained motivation and continual intention are necessary, but not sufficient for behavior change [24, 25, 39].

Theoretical underpinning for SystemCHANGE™ have been detailed elsewhere [38] but a brief overview is provided here. Grounded in the socioecological model of Brofenbrenner, [40, 41] SystemCHANGE™ focuses on the micro level systems of face-to-face influences on MA in the person’s family, work, and social circles, and also on the meso level which consists of the individual’s interrelated micro level systems. Within this framework, SystemCHANGE™ supports patient-designed, interventionist-guided, small experiments using Deming’s Plan-Do-Check-Act cycle [42].

SystemCHANGE™ interventions have increased and maintained physical exercise, [23, 24] reduced sleep disorders, [22] reduced stress, [43] lowered asthma attacks, [44] improved eating behaviors, [45] and enhanced care of those with hypertension [46]. At the micro and meso level, our recent systematic review of personal system level interventions documents potential for improving difficult-to-change behaviors such as MA [47]. The focus of this study involves implementing the SystemCHANGE™ intervention with the patient at the micro and meso personal level, not at the exo or large system or community level.

### Study sample and setting

Participants are being recruited from two kidney transplant centers. The transplant centers’ staff (transplant nurses and social workers) are using a computer-generated list of random numbers provided by the study biostatistician to randomly select 190 potential Pps from a list of transplant patients cared for at their respective transplant center (95 from University of Missouri [MU] and 95 from University of Tennessee [UT]). Staff telephone identified patients and ask if they are willing to have a RA contact them to discuss possible participation in a study. If they are willing to be called, the RA will contact them by telephone to review the study. If the patient agrees to participate the electronic medication monitoring cap and diary will be mailed to them, the cognitive screening exam administered, and demographic information gathered.

### Eligibility and exclusions

Adult kidney transplant recipients meeting the following criteria will be included: 1) age 18 years or older, 2) prescribed at least 1 immunosuppressive medication taken twice a day, 3) functioning kidney transplant (not on dialysis), 4) has received a kidney-only transplant, 5) agreement from the transplant physician and nephrologist that the individual is able to participate in the study, 6) able to speak, hear, and understand English as determined by the ability to participate and comprehend conversation about potential inclusion in the study, 7) able to open an electronic medication monitoring cap as assessed by the RA asking if there is any problem with opening pill bottle caps, 8) able to administer immunosuppressive medications to self, 9) has a telephone or has access to a telephone, 10) has no cognitive impairment as determined by a score of 4 or greater on the 6-item Telephone Mental Status Screen Derived from the Mini-Mental Status Exam, 11) has no other diagnoses that may shorten life span, such as metastatic cancer, 12) is not currently hospitalized, 13) receives post-transplant care by the Missouri or Tennessee transplant programs. Patients who have had their transplants for various lengths of time are being recruited because the variable ‘time since transplant’ has been shown to be an unreliable predictor of medication nonadherence [42–44]. Patients receiving other types of transplants are being excluded from the study because MA varies between transplant types [8]. Patients who receive a kidney re-transplant are included since medication nonadherence also occurs in this subset of kidney transplant recipients [43, 45]. The few kidney transplant recipients who participated in the pilot intervention study are excluded from this study.

### Randomization after allocation procedure

We will employ stratified randomization, which is directed by a biostatistician. Participants with a MA score <.85 will be randomly assigned to either the treatment or control group by a computer-generated block randomization scheme. We will also stratify by moderate (84-70 %) and low (<70 %) adherence to maintain balance between the treatment and attention-control groups. Participant number is sequentially assigned in the order in which individuals are consented. If a Pp drops out in the intervention phase, the next enrolled Pp is assigned to the same group (treatment or attention-control) as the drop out was assigned. Although requiring RAs from both study groups to be available at study enrollments appears inefficient, in our experience it is a great advantage to engage new enrollees immediately in our treatment protocol and thereby eliminate potential attrition between randomization and the first intervention or control session.

### Blinding

All study personnel except the biostatistician are remaining blind to the group assigned until after eligibility is determined. Afterward, the PIs discloses the assigned Pp code and provide their information to the appropriate RA for the assigned intervention to begin.

### Development of the SystemCHANGE™ intervention

Our previous qualitative studies of medication self-management in adults and older adults indicate environmental structure and routines are important for success [46, 47]. Strategies include maintaining routines (habits and linking medication taking with other behaviors), reminder methods (cues, alarms, pillboxes, and medication location), obtaining medications (pharmacy routines) and involving a person who supports the medication taking environment. Consequently, these strategies are incorporated into the SystemCHANGE™ intervention to enhance medication self-management which has traditionally been absent from transplant patient education [48].

SystemCHANGE™ is delivered in various formats (group versus individual) over different time frames (one time to 12 weeks), and in several locations (home versus community center) [49]. We are delivering the SystemCHANGE™ intervention in the kidney transplant recipients’ homes and over the telephone since many travel long distances to a transplant center. This delivery approach facilitates the sustainability of the intervention.

The baseline SystemCHANGE™ home visit is approximately 1 hour and 20 minutes in length. Table 1 provides an overview of the first step of the SystemCHANGE™ intervention delivered during the home visit. During the second step, which is delivered over the telephone 2 weeks after the home visit, the RA and Pp recount the Pp’s discussion with the important person(s) and the selected environmental solution identified during the home visit. The RA asks the Pp to identify a date to implement the solution and encourages the Pp to continue using the electronic medication monitor. They schedule a time to speak by telephone in 1 month to review the electronic medication monitor report and evaluate progress.

**Table 1:** SystemCHANGE^TM^ Intervention-home visit

Topic	Content
Introduction of SystemCHANGE^TM^	Overview-challenges of medication taking (takes time, not your fault, should not have to try harder, medications should be in the right place at the right time, habits and routines are key to success), focus on routines and conducting small experiments to improve medication taking, review 4 steps of SystemCHANGE^TM^, and discuss how RA will guide Pp through the process of improvement.
Review of electronic medication self-monitoring report	RA guides Pp’s review of MEMS report details; Pp’s personal MEMS report reviewed from screening phase.
Goal setting	RA discusses Pp’s medication taking goals; encouraged to have goal of 100 % medication adherence.
Determining Process Owners	RA assists Pp in identifying important people in medication taking process using Important People worksheet.
Lifestyle Routines	RA assist Pp in identifying lifestyle routines using the Life Routines worksheet.
Cycles	RA assists Pp in identifying cyclical nature of daily, weekly and monthly routines using the Cycles worksheet.
Possible Solutions for Change	RA and Pp collaboratively consider possible environmental changes to enhance medication taking routines. Participant scores ideas on Possible Solutions Scale.
Storyboard	RA encourages Pp to post MEMS report as a storyboard for success.

During the next phase of the study, step 3, medication taking goals and the “small experiments” are evaluated. This occurs each month during a telephone call by the RA to the Pp. The RA mails the electronic medication monitor report to the Pp prior to the call during which the RA asks the Pp “Tell me what you are learning about medication taking. How to you think changes you have made to routines are changing your medication taking? Tell us about any other changes to medication taking routines that you feel need to be made.” If adherence is the same or worse as before, the RA encourages the Pp to try another solution from the Possible Solutions list completed during the home visit. If medication taking is improving, the RA encourages the Pp to continue that approach. The RA reminds the Pp to share progress through a storyboard by displaying the electronic medication monitoring reports in a prominent location, such as the refrigerator.

After month 6 of the intervention phase, the RA closes the intervention by discussing the Pp’s improvements. The RA encourages the Pp to continue using the electronic medication monitor and diary for the next 6 months during the maintenance phase.

### Attention-Control intervention

The 6-month attention-control intervention involves a home visit and monthly phone calls at the same intervals as the intervention group (at attention-control months 1, 2, 3, 4, 5, and 6). Rather than the SystemCHANGE™ intervention, the attention-control Pps receive educational materials developed by the International Transplant Nurses Society that address healthy living after transplant [50]. The RA calls Pps at 1, 2, 3, 4, 5 and 6 months to review the brochure information and answer any questions about it. Time, interval, frequency, and setting are all exactly the same for the intervention and control groups. If attention-control Pps raise questions about medications or medication-taking, the RA directs them to discuss them with their transplant team contact person.

At the end of month 6 of the intervention phase, the RA closes the control by discussing the healthy post-transplant information the Pp reviewed during the previous 6 months. The RA encourages Pps to continue using the electronic medication monitor and diary for the next 6 months during maintenance phase.

### Treatment fidelity

A detailed procedure manual provides specific information about every facet of the interventions. To ensure RA fidelity to the intervention and control arms, a Fidelity Protocol Checklist is used during all Pp encounters where key elements of the protocol are documented including number of intervention sessions, session duration, length of time between sessions, and intervention steps (e.g. greeting the Pp, MEMS review, use of intervention forms). Each element is rated as completed, partially completed, not completed, or N/A. Field notes are documented for every encounter, which could include Pp’s body language, environmental issues (e.g. temperature, noise), and presence of others in the home. Field notes for telephone contacts include background noise, telephone line distortion, and any difficulty hearing by RA or Pp.

### Training the research assistants who deliver the intervention

An expert in SystemCHANGE™ delivers content training to RAs who are baccalaureate-prepared RNs at the study recruitment sites. To preserve intervention integrity, simulation and role play are used until the RAs are applying the protocol consistently, as judged by the expert using the SystemCHANGE™ protocol checklist. In addition to teaching RAs SystemCHANGE™ principles and steps, the expert guides RAs as they practice using the study protocol and protocol checklist on a sample of Pps. To ensure the highest level of RA protocol knowledge and skills, training sessions also include role playing of disruptive situations for both interventions, and delivering both intervention for a different behavior change such as exercise or diet. SystemCHANGE™ RAs are trained separately from the control RAs. The expert provides RAs feedback on performance, and RAs retrain as necessary until they have achieved 100 % intervention protocol integrity.

### Primary outcome - medication adherence

Table 2 provides an overview of study measures and outcomes. The MA calculation method has been previously described but will be briefly described here [41]. A 0.5 is assigned if the dose of the immunosuppressive medication is taken within a 3-hour window (+/-1.5 hours of the prescribed time); 0.25 is assigned if the dose of the immunosuppressive medication is not within the 3-hour window but is taken within a 12-hour window (+/-6 hours of the prescribed time), and 0 is assigned if the dose of the immunosuppressive medication is not taken within a 12-hour window (+/-6 hours of the prescribed time, i.e., if the dose was missed). On each day, an individual is assigned a score of 0, 0.25, 0.50, 0.75 or 1 points (p. 526).

**Table 2:** Study outcomes and measures

	Screening	Intervention	Maintenance
*Inclusion Criteria*			
6-item telephone measure of cognition	√		
Primary Outcome			
Medication adherence–electronic medication monitoring	√	√	√
Secondary Outcomes			
Creatinine	√	√	√
Blood urea nitrogen	√	√	√
Acute rejection	√	√	√
Chronic rejection	√	√	√
Infection	√	√	√
Death	√	√	√
*Potential Mediators*			
Social Support		√	√
Perceived Health		√	√
Personal Systems Thinking		√	√
*Background*/*Moderating Variables*			
Medical History	√		
Demographics (ethnicity)	√		
Level of Medication Nonadherence	√		
Pillbox use	√		
*Health care costs*			
Hospitalizations, clinic, observation, and ER visits	√	√	√

The primary outcome is medication adherence measured by the MEMSCap™ Medication Event Monitoring System SmartCap® (WestRock, USA & Switzerland). The system is comprised of two parts: a standard plastic vial with a threaded opening and the SmartCap® which has an LCD readout that displays the number of doses taken in the past 24 hours and the hours elapsed since last dosing. A micro-electronic circuit in the SmartCap® registers the date and time when the top is opened and closed to create a medication “event”. Time-stamped medication events stored in the MEMS® 6 can be transferred at any time through the MEMSCap™ Wireless Reader to medAmigo. The medAmigo portal is a web-based application used to securely download and centrally store medication dosing history (www.medAmigo.com). MedAmigo performs the MA score calculations. Although no gold standard measure exists for MA, most researchers consider electronic monitoring caps the best method. It is the only adherence measure that can accurately assess this variable, as recall memory is unlikely to be accurate enough for self-reports to provide valid data regarding the exact timing of doses over a period of time. The batteries for the MEMS 6 have a 36 month life and can store up to 3800 medication events. This battery life has been shown to be more than sufficient for capturing 12 months of medication taking activity [51]. The MEMS has been shown to be reliable in temperatures from -20 °C to 70 °C and in up to 95 % humidity [52], are accurate to within 2 minutes per month, and have a reported 2 % failure rate [52, 53]. The ability of electronic monitoring to provide a precise assessment of dosage timing is particularly advantageous as studies are beginning to emerge that reveal the importance of the interval between doses in explaining the relationship between adherence and clinical outcomes in other chronic illnesses [54, 55].

Both intervention and attention-control groups use MEMS for 6 months after the 3-month screening phase. This length of time allows adequate time to capture changes in medication-taking behavior [56]. One twice-daily prescribed medication are monitored because previous research has indicated that monitoring a second medication does not provide additional MA information [53]. The monitored immunosuppressive medication is randomly selected by the RA. Random selection of the monitored immunosuppressive medication occurs as follows: The RA numbers all of the twice-daily administered immunosuppressive medications on the Demographics Form. Most Pps will take two immunosuppressive medications twice daily. In this case, the RA will flip a coin to determine which medication will be placed in the MEMS bottle. In the event that the patient has greater than two immunosuppressive medications taken twice daily, the RA will enter the number into a random numbers generator and have the Pp monitor the randomly selected immunosuppressive medication.

Pps are trained on use of the MEMS cap by the RAs. They are instructed to only remove the randomly selected immunosuppressive medication from the MEMS cap and bottle. If a pillbox is used for medications, colored disks/“Tic-Tacs” are placed in the pillbox to remind the Pp to ingest the medication from the MEMS bottle. During the screening phase, at weeks 1 and 8, the RAs telephones the Pp to ask if they are using the MEMS and MEMS diary and if they have any questions about using them. Those Pps who do use a pillbox are asked if they are using the “Tic-Tacs” to remind them to ingest from the MEMS bottle. If there are any deviations from the procedure, the RAs will re-train the Pps.

Beginning the day after the intervention Pps are instructed how to use the cap. Cap openings will be recorded and a cumulative medication taking record generated. This record is sent to the SystemCHANGE™ intervention Pps and reviewed with them at baseline and months 1, 2, 3, 4, 5, and 6. These reports contain a summary report with graphic representation of individual bottle openings and closings within the established time window of +/-1.5 hours, the hours elapsed since the previous opening, missed doses, and drug holidays. The attention-control intervention group will continue to use the MEMS but will not be sent a report since this is the “Study” step of the SystemCHANGE™ intervention.

Participants mail the MEMS diary to the RA to document any accidental cap openings, openings when no medication was ingested, (e.g. when refilling MEMS bottle), and early openings when a medication was removed early to be administered later (pocketing a dose), but on time, (e.g. clinic appointments). As in our preliminary work, we will correct MEMS cap data using the MEMS diary. The diary successfully corrects any invalid data from MEMS opening when medications were not ingested or were ingested at a time different from the time the MEMS was opened [57]. After these corrections, we assume that each cap removal represents the patient ingesting one dose of the prescribed immunosuppressant. To enhance accuracy, Pps are trained on use of the MEMS diary. Pps are given specific examples of when the diary should and should not be used. They are trained to store the diary with the MEMS bottle. Training continues until the Pp achieves 100 % accuracy using the MEMS diary with 4 MEMS diary test scenarios (i.e., accidental opening, early opening [pocketing dose], opened but no medication administered, and diary storage). This approach to using a MEMS diary to correct adherence data has been validated in several previous research studies [19–43, 53, 57].

### Additional outcomes

The following clinical outcomes will be collected retrospectively for all three phases: Blood creatinine, BUN level, acute and chronic rejection, infection, health-related quality of life and death from the medical record and from primary data collection. Acute and chronic rejection episodes will include those that are biopsy-proven and/or medically treated (3-day dose of intravenous prednisone) as such. Infection episodes will include those in which the blood, sputum, and/or urine culture is positive for an abnormal organism. Deaths will be reported from the transplant team.

### Cost-effectiveness

The primary endpoint of cost-effectiveness measures will be the incremental cost-effectiveness ratio (ICER) of the SystemCHANGE™ intervention relative to the attention-control, which assesses the incremental cost per health-related quality-adjusted life year gained. The perspective of cost-effectiveness is a third-party payer. A micro-costing approach will be used to measure the intervention’s resource use, based on a log of resource use for each intervention. The resources used for the delivery intervention in the interventional and the control group will be tracked over the study period. The Pps will track the type and quantity of medical services consumed (doctor’s office, clinic, hospital, medication). The unit cost of personnel time will be based on actual hourly salary rates and fringe benefits. Unit costs of each hospitalization, ER visit, clinic visit, and physician fee will be estimated based on Medicare’s average reimbursement rate. The unit cost of medication will be estimated from the average wholesale price plus the dispensation fee of 2 %. To determine the number of quality-adjusted life years over the observational period, the weight will be multiplied by the number of days in the observational period. All cost measures will be adjusted to the constant U.S. dollar. Sensitivity analyses will examine key parameters that may affect ICERs.

### Potential moderators and mediators

Perceived health status, a potential moderator, will be measured by one question, “In general, how would you say your health is?” Respondents select excellent, very good, good, fair, or poor. Perceived health status reflects people’s overall perception of their health, including both physical and psychological dimensions [58]. The question has good reliability and validity [58]. Ethnicity will also be examined as a moderator.

Potential mediators are examined including social support and systems-thinking. We measure social support using the Social Support Appraisals Index, a 23-item self-administered, self-report scale measuring the degree to which a person feels cared for, respected, and involved with family and friends [59]. Respondents strongly agree, agree, disagree, or strongly disagree with each statement. Total scores range from 23 to 92. After reversing the negatively stated items, low scores indicate high levels of support. Typically, subscale scores for family and friends are calculated. The instrument has been used with adult kidney transplant recipients [43, 60]. Data from 10 samples indicate that the scale had good internal reliability, with Cronbach’s alphas ranging from .80 to .90 [59]. The scale also showed stability over a six-week interval, with reliability scores of .80. Convergent validity has been demonstrated with significant associations to seven other appraisal measures. Moreover, adequate concurrent, and divergent validity with other perceived support measures was demonstrated.

Personal Systems Thinking will be measured by Systems Thinking Survey (adapted for patients), a 20-item scale using a 5-point Likert response scale developed by Drs. Dolansky and Moore. The scale measures perceptions of personal system behaviors [61]. It has good reliability and construct and discriminate validity [62]. Test-retest was 0.74 and Cronbach’s Alpha was 0.89 [62]. The tool discriminated between those receiving high and low or no SystemCHANGE training (*p*=.05 and .01, respectively).

### Statistical analysis

Sample size and power calculations are based on comparing expected change in medication adherence rate of patients in each group at six months - an expected adherence mean difference of 10 % based on our pilot study findings and the literature. We use an alpha of .05 and provide for 90 % power to detect indicated effect sizes in this two-arm randomized study. An effect size difference of 70 % is based on a conservative estimate of our pilot work. A sample size of 46 older KT recipients per group (final total sample 92) will meet these assumptions and provide sufficient power. Recruitment and retention rates are calculated from our pilot study, other adherence studies at the same sites, and are documented in one similar RCT adherence study in older KT recipients [63, 64]. We selected an adherence rate of 85 % to divide the adherers from the non-adherers based upon our preliminary work describing 4 clusters of KT medication adherers: those who (1) take medications on time (1.0-.85 MA rate), (2) take medications on time with late/missed doses (.84-.70 MA rate), (3) rarely take medications on time and who were late with morning and/or evening doses (.69-.20 MA rate), and (4) missed many doses (<.20). Even minor deviations in dosing adherence lead to poor outcomes, though no studies have determined the criterion adherence “dose” that distinguishes good and poor outcomes.

Appropriate descriptive analyses will be performed to examine distributional characteristics for collected measures, as well as to summarize changes over time as a function of group assignment. During this initial phase, we will explore bivariate relationships among primary and secondary outcome measures and variables thought to affect medication adherence. In addition, we will conduct analyses to determine whether the randomly assigned groups are equivalent at the start of the study on the demographic and other measures collected at baseline. Before hypothesis-testing analyses are conducted, exploratory analyses will be performed to examine the effect of various mediators and moderators on the relationship between intervention, adherence, and clinical outcome. The results of these analyses will determine what additional variables will be incorporated in the subsequent hypothesis testing (e.g., analysis of covariance).

Our primary analysis assesses whether the SystemCHANGE™ intervention is more effective than the attention-control intervention in increasing MA in adult kidney transplant recipients at the completion of the 6-month intervention and 6-month maintenance phases. We hypothesize that adult kidney transplant recipients receiving the SystemCHANGE™ intervention will have higher immunosuppressive MA rates than the attention-control group at the completion of intervention and maintenance phases. Since rate responses will most likely violate the normality assumption, the non-parametric method, Mann- Whitney test, will be used for comparing the two groups. However if the normal assumption is satisfied through transformation or as raw data measures, t-test will be applied for group comparison. Possible covariates resulting from demographic data and screening phase MA will be included in the analysis to adjust for possible bias.

Our secondary analysis assesses the MA patterns in both the SystemCHANGE™ and attention-control groups. Specifically we are interested in determining when the intervention becomes effective (e.g., what “dose” is needed) and the pattern of decay in MA over time in both groups. The dependent variable for these research questions are the repeated measurements of immunosuppressive MA rates at 12 time points [i.e. 1, 2, 3, 4, 5, 6, 7, 8, 9, 10, 11 and 12 months] and the independent variable is group assignment and time effect. Poisson regression analysis will be used for these questions. Proc Nlmixed procedure in SAS will be used for Poisson regression modeling. In order to answer the hypothesis we will test for group-by-time interaction to test if the two groups have different time profiles for MA or not. Possible covariates resulting from demographic data and screening phase MA will be included in the model to adjust for possible bias. Repeated measures from the same Pp will be accounted for using a random effect in the model.

Our exploratory analyses focuses on three aims: 1) to determine whether the SystemCHANGE™ intervention is more effective than the attention-control intervention in decreasing poor health outcomes (e.g. increasing creatinine/BUN, infection, acute/chronic rejection, graft loss, death), 2) to evaluate the role of potential mediators (social support, and systems-thinking) and moderators (ethnicity perceived health and level of medication nonadherence) of MA and health outcomes in adult kidney transplant recipients receiving the SystemCHANGE™ intervention, and 3) to determine if the SystemCHANGE™ intervention is cost-effective.

We expect to observe lower levels of poor health outcomes in the SystemCHANGE™ group as compared to the control group. The dependent variables are the dichotomous outcomes such as, infection, acute and chronic rejection, graft loss, and death and numeric outcomes such as creatinine, and BUN.

The independent variable is group assignment. Chi-square tests will be applied to estimate and test the relationships between dichotomous outcome variables and the independent variable. For continuous outcomes variables t test or Mann-Whitney test will be conducted to test for group effect depending on the satisfaction of normality assumption.

Our secondary exploratory aim is to evaluate the role of potential mediators and moderators of MA and health outcomes in adult kidney transplant recipients receiving the SystemCHANGE™ intervention and recipients receiving the attention-control intervention. We hypothesize that exploring potential mediators and moderators in the analyses will enhance the interpretation of treatment effect on MA. To test for mediator effect, the dependent variable is MA and the independent variable is treatment group. Potential mediators are social support and systems thinking. Poisson regression will be applied to estimate the mediator effect [65] and the Sobel test [66] will be used to test if the mediator effect is significant. Changes in the variance with mediator in the models will be estimated and reported as part of the analysis results. To test for moderator effect, the dependent variable is MA and the independent variable is treatment group. Potential mediators are ethnicity group, perceived health and MA level (different strata). Poisson regression will be applied to estimate and test for possible moderator effect through interaction terms between the independent variable and moderator [65].

Our third exploratory aim is to determine if the SystemCHANGE™ intervention is cost-effective. Our hypothesis is the cost for the SystemCHANGE™ intervention will be less than the cost for the attention-control intervention. To determine the cost-effectiveness of the SystemCHANGE™ intervention compared to the attention-control intervention, both intervention and resource use costs will be evaluated and compared to MA change. A cost-effectiveness analysis will be performed at the end of the intervention period and again at the end of the maintenance period. If there is no treatment effect, a cost-analysis will not be performed. The analysis performed at the end of the maintenance period will be cumulative, incorporating costs and benefits incurred throughout the project. A cost-effectiveness analysis, performed at the end of the maintenance period (calculated for both the intervention and maintenance periods), will evaluate both intervention and control, and resource use costs which will be compared to adherence change. The sum of the total intervention and control costs and resource use costs will be the numerators for testing this hypothesis. The change in adherence (from baseline to end of intervention [or end of maintenance] period) will be the denominator. We will identify all direct intervention costs related to the intervention and the control (planning, designing, and implementation of each intervention, personnel, supplies, travel, and equipment). We will identify resource use costs (hospitalizations, clinic, observation, and ER visits) for both groups. Resource use costs will be obtained from publically available data, e.g. Hospital Compare, Hospital Stats, H-CUP. The DRG will be obtained with a conversion rate and then adjusted by hospital specific information, e.g. academic, location.

## Discussion

This is the first fully-powered, randomized, controlled trial to determine the effectiveness of a SystemCHANGE™ intervention in increasing medication adherence in adult kidney transplant recipients. The sample population is adult kidney transplantation recipients. The results of this study can potentially impact the science of adherence research radically for an extended period of time. Adherence intervention research is languishing with modest results for over 35 years. Medication adherence intervention studies traditionally target individuals’ characteristics, such as knowledge, attitudes, and beliefs. They are marginally effective for those with acute and chronic illnesses [14–16, 67] and ineffective for adult transplant recipients [19, 20]. Clinicians are frustrated by their inability to offer patients effective medication adherence interventions. Patients are tired of being blamed for medication nonadherence. We need effective interventions immediately to prevent loss of implanted kidneys, but also to make additional kidneys available to those waiting for transplants by reducing the number of transplant recipients who must rejoin the transplant waiting list because medication non-adherence caused their kidney transplant to fail. The impact of the intervention on other health outcomes and healthcare charges must be examined. We need a paradigm shift from focusing on medication adherence patient knowledge, attitudes, beliefs, and behavioral skills to the patient’s personal environment and daily routines that influence MA [68].

If this SystemCHANGE™ intervention is found to be effective in kidney transplant patients, other chronically ill populations known to have medication nonadherence (those with hypertension, diabetes, TB, asthma, epilepsy) may immediately benefit from this approach while trials are conducted to confirm findings across populations. The obdurate problem of medication nonadherence is rampant. Scientists’ medication nonadherence interventions will move from focusing primarily on interventions to change knowledge, attitudes, and beliefs and instead are focusing on changing the personal environment and shaping daily routines. Clinicians will cease blaming the patient for the inability to adhere to the prescription, but will, within the SystemCHANGE™ framework, support patient-designed, interventionist-guided, small experiments using Deming’s Plan-Do-Check-Act cycle [69] Our pilot study results indicate that the SystemCHANGE™ intervention “dose” may be effective immediately. If these results are supported in this larger study, clinicians could quickly and easily deliver the intervention in the hospital or clinic setting at a reasonable cost. Electronic monitoring systems are constantly improving. Wireless systems are streamlining electronic monitoring of medications, which further enhances the ease of delivery. The findings of this study may also prompt researchers to explore SystemCHANGE™ approaches to changing other important public health problems arising from human behavior (e.g. smoking).

In conclusion, each year, 35.6 kidney transplant recipients per 100 are non-adherent with their medications, which is the primary cause of post-transplant morbidity [8]. Thus, the need for effective interventions is compelling: Decreasing transplant complications from medication nonadherence will reduce costs and make additional kidneys available to those waiting for transplants by reducing the number of kidney transplant recipients who must rejoin the organ list. This project builds on our research team’s previous adherence work, including a SystemCHANGE™ intervention pilot study that addresses Healthy People 2020 initiatives of reducing chronic kidney disease complications, disability, death, and costs by optimizing transplant medication adherence and increasing the number of patients who receive a transplant [70]. Evidence suggests that a significant gap exists in the medication adherence intervention literature – a lack of changing the personal environment that either hinders or augments medication adherence. This study addresses that gap in that it is the first to evaluate a SystemCHANGE™ intervention to enhance medication adherence in kidney transplant patients in a fully powered study.
